# Complex non-*falciparum* transmission and widespread insecticide resistance in Penka-Michel, a hyperendemic malaria focus of the Cameroon highlands

**DOI:** 10.21203/rs.3.rs-9488336/v1

**Published:** 2026-05-25

**Authors:** Belinda Claire Kiam, Ibrahima Ibrahima, Jean Arthur Mbida Mbida, Aline Gaëlle Bouopda-Tuedom, Luc Abate, Samuel J. White, Charlène Tina Nanssong-Vomo., Brigitte Fotso Tumamo, Isabelle Morlais, Lawrence Ayong, Jessica T. Lin, Innocent Mbulli Ali, Jonathan J. Juliano, Rhoel R. Dinglasan, Sandrine Eveline Nsango

**Affiliations:** 1 Faculty of Science, University of Douala, BP 24157, Douala, Cameroon; 2 Malaria Research Unit, Centre Pasteur du Cameroun, BP 1274, Yaounde, Cameroon; 3 MIVEGEC, Université Montpellier, IRD, CNRS, Montpellier, France; 4 Division of Infectious Diseases, Department of Medicine, School of Medicine, University of North Carolina, Chapel Hill, NC 27599, USA; 5 Department of Biochemistry, Faculty of Science, University of Dschang, B.P. 96 Dschang, Cameroon; 6 Department of Infectious Diseases & Immunology, College of Veterinary Medicine & Emerging Pathogens Institute, University of Florida, Gainesville, FL 32611, USA; 7 Faculty of Medicine and Pharmaceutical Sciences, University of Douala, P.O Box 2701, Douala, Cameroon; 8 Department of Biomedical Sciences, Faculty of Science, University of Bertoua, B.P 416, Bertoua, Cameroon

**Keywords:** Malaria, Transmission dynamics, *Anopheles*, insecticide resistance, *Plasmodium ovale* spp. Western Highland Cameroon

## Abstract

A 12-month longitudinal entomological study (September 2023-August 2024) in the Western Highlands of Cameroon (Penka-Michel) investigated malaria transmission dynamics and insecticide resistance markers, including knockdown resistance mutations (L1014F, N1575Y) and metabolic resistance genes (G119S-*Ace1*, E205D-*CYP6P3*, L119F-*GSTe2*, *CYP6P9a/b*). Malaria transmission has depended on the coexistence of three species: *Anopheles funestus* (43.7%), *Anopheles gambiae* (42.9%), and *Anopheles ziemanni* (13.5%). The site was hyperendemic with annual EIR of 839.5 infective-bites/human/night. *Plasmodium falciparum* predominated (70.2%), yet a significant non-*falciparum* burden was observed (*P. malariae*, 27.5%; *P. ovale*, 22.1%). *An. funestus* was the principal driver (IR=7.8%, EIR=0.9 infective bites/human/night), with *P. falciparum* and *P. ovale curtisi* peaking in the later rainy season (November), while *An. gambiae* (IR=5.4%; EIR=0.6 ib/h/n) vectored *P. malariae* (14.5%) primarily, followed by a sustained peak in the dry season (January). Widespread insecticide resistance was observed, with resistant homozygotes detected in 70% of infected mosquito samples. We observed associations of *L119F*-*GSTe2* mutations with *P. ovale curtisi* transmission (IRR=1.15, *p*=0.03), and *L1014F*-*kdr* with *P. malariae* (IRR=2.2, *p*=0.04). These dynamics highlight a complex multi-species system in which seasonal vector efficiency and insecticide resistance sustain hyperendemicity of the often-overlooked non-*falciparum* malaria species.

## Introduction

Malaria remains one of the most pressing public health threats in sub-Saharan Africa, with the majority of the burden concentrated in the WHO African Region. The disease accounted for more than 263 million cases and 597,000 deaths worldwide^1^. Cameroon is among the 29 high-burden, high-impact countries, contributing nearly 3% of global cases and 1.9% of malaria- related deaths^1^. Despite sustained control efforts, the country reported an estimated 2.9 million confirmed malaria cases and 1756 deaths in 2023^1,2^, with children under 5 years and pregnant women remaining the most vulnerable group^2^.

Malaria epidemiology in Cameroon is characterized by pronounced ecological heterogeneity, resulting in diverse transmission patterns across the country. While control efforts have traditionally focused on lowland forest and savanna areas, the highland regions of Western Cameroon represent a distinct eco-epidemiological zone where vector ecology, transmission dynamics, and disease burden differ markedly from those in lower-altitude settings^3,4^. Penka-Michel, located at approximately 1,500 m above sea level, exemplifies this highland environment: its undulating topography, valley systems, and microclimate conditions create a mosaic of permanent and transient mosquito breeding habitats that sustain malaria transmission despite relatively cooler temperatures^5^. Recent entomological surveys along an altitudinal gradient, including the Penka-Michel site, have revealed substantial *Anopheles* diversity, with *An. gambiae sensu lato* (s.l.), *An. funestus* s.l., and *An. ziemanni* predominating in these local vector populations^6^. *An. gambiae* s.l. exhibits its highest abundance in Penka-Michel, where human-biting rates (HBR) reach 45.3 bites/human/night, significantly higher than those recorded in neighboring lower-altitude sites such as Santchou (3.1) and Dschang (0.41)^6,7^. As a high transmission focus within the Western Highlands, Penka-Michel reported a local malaria prevalence of 19.7%, primarily driven by *Plasmodium falciparum*^7^. While non-*falciparum* species (*P. malariae* and *P. ovale*) persist at lower prevalence, the combination of high vector aggressiveness and stable transmission makes Penka-Michel a sentinel site for studying malaria persistence in this highland-specific ecological niche^6,7^.

Vector control is a cornerstone of malaria management in Cameroon, with reliance primarily on the large-scale deployment of long-lasting insecticidal treated nets (LLINs) and indoor residual spraying (IRS)^8^. While these interventions, combined with case management, have significantly reduced the malaria burden, their efficacy is increasingly threatened by the rapid spread of insecticide resistance in *An. gambiae* and *An. funestus* populations^9,10^. In Cameroon, both target-site mutations (such as *kdr* and *Ace*-1) and metabolic resistance mechanisms driven by the overexpression of detoxification enzymes, such as cytochrome P450s and glutathione S-transferases (e.g., the L119F-GSTe2 mutation), have been well documented^11–13^. These mechanisms not only confer high levels of resistance but also enhance vectorial capacity by increasing mosquito longevity and infectivity^11,14^. Genome-wide association studies in *An. funestus* populations across diverse eco-geographical settings in Cameroon have revealed strong signatures of positive selection at detoxification-related loci, closely associated with environmental and climatic variables, indicating that resistance evolution is shaped by local eco-climatic contexts^15^. This suggests that adaptive responses may differ fundamentally between highland and lowland populations. Consequently, granular molecular surveillance is essential to inform evidence-based resistance management, such as strategic insecticide rotation or the deployment of next-generation LLINs, to maintain the effectiveness of core control tools.

Despite these advances, important knowledge gaps persist. Longitudinal entomological and epidemiological data remain scarce for highland settings such as Penka-Michel, where the intersection of vector species composition, *Plasmodium* infection dynamics, and patterns of insecticide-resistance allele frequencies is poorly documented^3,8^. Furthermore, while climatic variables such as rainfall, humidity, and temperature are known to influence larval habitat and selection pressure, their integrated role in shaping the transmission of both *P. falciparum* and non-*falciparum* species in highland ecosystems remains under-investigated.

To address these gaps, this study undertakes a one-year entomological and molecular survey in Penka-Michel. The specific objectives are to (i) characterize the temporal composition and relative abundance of *Anopheles* species; (ii) assess vectorial capacity through measurements of biting behavior and *Plasmodium* infection rates; (iii) genotype key insecticide-resistance alleles, including *kdr* gene (*kdr*-L1014F, and N1575Y) and metabolic resistance genes (*Ace1-G119S*, *E205D*-*CYP6P3*, *L119F-GSTe2*, and *CYP6P9a/b*). in local vector populations, and (iv) examine relationships between climatic variables (rainfall, temperature, humidity, and wind speed) and vector abundance, transmission indices, and resistance allele dynamics. By integrating entomological, molecular, and climatic datasets, this study aims to generate robust, policy-relevant evidence to guide climate-responsive, locally adapted vector control strategies and to elucidate the role of non-*falciparum* malaria species in transmission dynamics, which remains insufficiently addressed in existing control frameworks in the highland regions of Cameroon.

## Methods

### Description of study area

**Figure 1:**
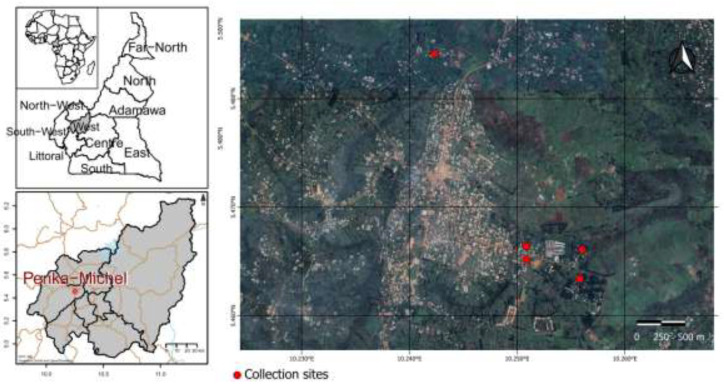
Map showing Penka-Michel collection sites, Western Highlands, Cameroon.

The study was conducted in Penka-Michel (5°28’N; 10°15’E) (Fig. 1), one of the five subdivisions of the Menoua Department in western Cameroon. The area covers 262 km^2^ and has an estimated population of 98,229 residents. Situated at 1,500 meters above sea level, it is part of the central Bamileke highland zone and is characterized by a relatively flat plateau punctuated by a few convex-concave hills (8–12% slopes) and narrow, shallow valleys^5^. The climate is sub-equatorial and altitude-dependent. It is marked by a long rainy season from mid-March to mid-November, with peak rainfall between August and September, reaching up to 345.1 mm, followed by a short dry season. Temperatures remain relatively low and stable throughout the year, with an annual variation of approximately 2.6°C, comparable to climate conditions recorded in the nearby town of Dschang^5^.

Housing in Penka-Michel consists predominantly of earth-brick or cement-coated dwellings with aluminum or straw roofing. These dwellings are situated within a peri-urban landscape (Fig. 1), characterized by residential clusters interspersed with intensive agricultural plots. The proximity of households to localized drainage and small-scale cultivation areas provides suitable larval habitats for *An. gambiae* s.l. and *An. funestus* s.l., which constitute the principal malaria vectors in the area^6^. While historically considered a low-transmission zone, recent epidemiological studies at these sites revealed a *falciparum* malaria prevalence of 19.7%^7^.

### Meteorological Data Acquisition

Meteorological data for the study period (September 2023 to August 2024) was collected through remote sensing to create a comprehensive set of climate variables. The data was obtained from NASA’s POWER (Prediction of Worldwide Energy Resources) satellite portal^16^. The variables extracted included total daily precipitation, daily average relative humidity, daily temperature (average, maximum, and minimum), and average wind speed. These data were used to assess temporal associations among climatic conditions, vector abundance, malaria transmission indices, and insecticide resistance dynamics.

### Field sampling of adult mosquitoes

Adult mosquito collections were conducted monthly over one year, from September 2023 to August 2024, in the Penka-Michel locality. Household selection was guided by a previous cross-sectional survey^6^, which had identified these locations as representative of local malaria transmission patterns.

Each month, collections were performed over two consecutive nights in five selected households, from 6 p.m. to 9 a.m., both indoors (living room or bedroom) and outdoors (in a designated zone). In each household, two trained volunteers collected host-seeking mosquitoes as they landed on their exposed lower limbs. Each volunteer worked 15-hour shifts and alternated between indoor and outdoor stations every two hours to minimize bias linked to individual attractiveness or collection efficiency. This design resulted in 20 human nights of collection per household, totaling 152 human-nights over the entire study period.

### Mosquito identification

Mosquitoes were initially sorted morphologically by genus (*Culex, Anopheles, Aedes, Mansonia*, and others) using standard identification keys^17^. *Anopheles* specimens were subsequently dissected, with the head-thorax separated from the abdomen, and each part individually preserved in Eppendorf tubes containing silica gel for molecular analysis. Genomic DNA was extracted from the head-thorax and legs using a 2% CTAB (cetyltrimethylammonium bromide) according to Collins et al.^18^ and DNA concentrations were measured using a NanoDrop^™^ spectrophotometer (ThermoScientific, Wilmington, USA). Species identification within the *Anopheles gambiae sensu lato* (s.l.) and the *Anopheles funestus* s.l. was performed using a multiplex PCR targeting the ribosomal DNA intergenic spacer (rDNA IGS) and the internal transcribed spacer 2 (ITS2), respectively^19,20^. For *Anopheles gambiae* s.l., restriction fragment length polymorphism (RFLP) analysis was subsequently carried out to differentiate sibling species^19^.

### Ovary dissection

Ovaries from approximately 20% of the collected unfed *Anopheles* mosquitoes were dissected in the field following capture to determine parity. Dissections were performed on sterile microscope slides using distilled water under a binocular loupe, and the preparations were examined under a binocular microscope at 10X magnification. Ovaries were classified as nulliparous (tightly coiled tracheolar skeins) or parous (loosely coiled tracheoles). All dissected mosquitoes were individually preserved in tubes containing silica gel for Plasmodium detection analyses.

### Molecular detection and differentiation of *Plasmodium* spp. and *P. ovale* subspecies

Each month, a subsample of genomic DNA extracts from the head-thorax of each *Anopheles* species was screened for *Plasmodium* infection using a quantitative real-time PCR (qPCR) following the protocol of Mangold et al.^21^. The assay targeted a polymorphic region of the 18S small subunit ribosomal RNA (18S rRNA) gene and was performed over 45 amplification cycles with HOT FIREPol^®^ Evagreen^®^ qPCR Mix Plus (ROX), 5x (Solis BioDyne). This approach enabled the differentiation of *P. falciparum, P. malariae, P. vivax*, and *P. ovale* sp. (**Additional file 1: Table S1**).

All samples identified as positive for *P. ovale* sp. or *P. vivax* sp. were subsequently confirmed using species-specific TaqMan real-time PCR assays, performed over 45 cycles. The assays were conducted according to the protocols described by Mitchell et *al*.^22^ and Brazeau et *al*.^23^, respectively, using 2X TaqMan Master mix. (**Additional file 1: Table S1**)

Following confirmation, *P. ovale* positive samples were further analyzed to differentiate *P. ovale curtisi* (*Poc*) from *P. ovale wallikeri* (*Pow*). This discrimination was performed using the protocol described by Potlapalli et al.^24^. The cycle threshold (Ct) thresholds were determined across a range of target plasmid copy concentrations (**Additional file 1: Table S1**). All reactions were performed using a Bio-Rad CFX Connect Real-Time PCR Detection System.

### Genotyping of resistance markers in *Anopheles* mosquitoes

To monitor insecticide resistance status of anopheline vectors, a monthly subsample of approximately 100 (distributed at 50 of the dry and rainy seasons) *Anopheles* mosquitoes per species was genotyped for key target-site and metabolic resistance markers. Table 1 summarizes the key insecticide resistance markers screened and their associated insecticide classes. The supplementary table provides an overview of primer sequences, thermal cycling conditions, and comprehensive reference sequences for each molecular assay (**Additional file 1: Table S2)**.

### Data analysis

All statistical analyses were conducted using R version 4.4.3 (R Core Team, 2025), with the RStudio interface (Posit Team, 2024). Statistical significance was set at *p*<0.05. Descriptive statistics including mean, proportions, standard deviation (SD) and 95% confidence intervals (CI) were used to summarize entomological parameters (vector diversity, relative abundance, biting rate, biting cycle and behavior, parity rate, daily survival rate, life expectancy, infection rate (IR), and entomological inoculation rate (EIR)), molecular data (mutation frequencies), and meteorological data (rainfall, temperature, relative humidity, and wind speed). Mean *Anopheles* mosquito density (D) was calculated as D = (Number of mosquitoes of each species/Number of houses)/Number of collection nights.

To evaluate monthly and seasonal variations in entomological parameters, two-way ANOVA was applied, followed by Tukey’s Honest Significant Difference (HSD) test for multiple comparisons. When normality assumptions were not met, Kruskal-Wallis tests were followed by Dunn’s post-hoc test for pairwise comparisons. Chi-square tests were applied for categorical data, including seasonal variations, *Plasmodium* infection rates, and EIR. A paired Wilcoxon signed-rank test was used to compare indoor vs. outdoor biting rates at the same collection points. Insecticide resistance mutation frequencies were compared seasonally using Chi-square and Fisher’s exact test, with Logistic regression applied to examine associations between mutation frequencies and environmental or entomological parameters.

To explore relationships between climatic factors, vector dynamics, and insecticide resistance, Spearman correlation coefficients were calculated between monthly meteorological variables, entomological parameters, and mutation frequencies. Generalized Linear Models (GLMs) with a Poisson distribution were used to assess the influence of climatic variables on vector dynamics and resistance mutation frequencies, and to investigate associations between resistance mutations and *Plasmodium* transmission.

## Results

### Mosquito abundance and species composition

A total of 6,371 mosquitoes were collected (September 2023 and August 2024), with *Anopheles* predominating (96.5%; n=6,152). Other genera included *Aedes* (1.9%, n=116, *Culex* (1.5%, n=96), *Mansonia* (0.08%, n=5), and *Coquillettidia* (0.03%, n=2) (**Additional file 1: Table S3**).

Morphological identification of the 6,152 female *Anopheles* revealed three species, *An. funestus* s.l. (43.7%, n=2,687), followed closely by *An. gambiae* s.l. (42.9%, n=2,636) and *An. ziemanni* (13.5%, n=829). Molecular analysis revealed *An. funestus* s.s. (90.7%, n=272/300) and *An. leesoni* (9.3%, n=28/300) within the *funestus* group, while the *An. gambiae* complex comprised *An. gambiae* s.s. (95.8%, n=365/381) and *An. coluzzii* (4.2%; n=16/381) (**Additional file: Fig. S1**).

**Figure 2:**
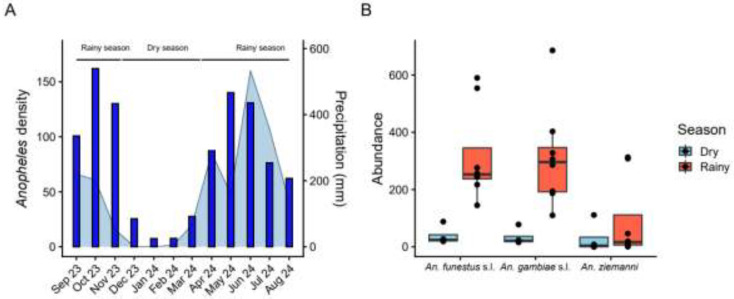
Monthly and seasonal variation of *Anopheles* mosquitoes during the study period. **A**: Monthly density variation associated with monthly precipitation (blue shaded area), **B:** *Anopheles* variation between seasons.

*Anopheles* abundance varied significantly throughout the year (ANOVA, F_1,11_=4.61, *p*<0.001), with a significantly higher mean density during the rainy season at 717.8 mosquitoes/month [95% CI: 711.6–723.9], compared to the dry season, at 102.5 mosquitoes/month [95% CI: 96.1–108.9] (*p*<0.01). Peak densities were observed during the late rainy season (October), reaching 162 mosquitoes/month, and in March (reaching 140 mosquitoes/month), at the end of the dry season (Fig. 2A).

Despite these seasonal fluctuations, the relative abundance of *An. gambiae* s.l. and *An. funestus* s.l. remained consistently high. *Anopheles gambiae* s.l. predominated during the late rainy season (September-November), averaging 472.3 mosquitoes/month [95% CI: 462.5–482.2], whereas *An. funestus* s.l. was more abundant during the early rainy season (May-August), with a mean of 409.5 mosquitoes/month [95% CI: 400.4–418.6], and the dry season (January-March), averaging 38.5 mosquitoes/month [95% CI: 29.4–47.6]. In contrast, *An. ziemanni* remained the least abundant species throughout the study period, peaking at the end of the rainy season (October-November), with a mean of 310.5 mosquitoes/month [95% CI: 310.2–310.8], and reaching minimal abundance at the beginning of the dry season (December) with 29.5 mosquitoes/month (Fig. 2B, **Additional file S1: Table S4**).

### Biting behavior, human exposure, and activity patterns of *Anopheles* mosquitoes

**Figure 3:**
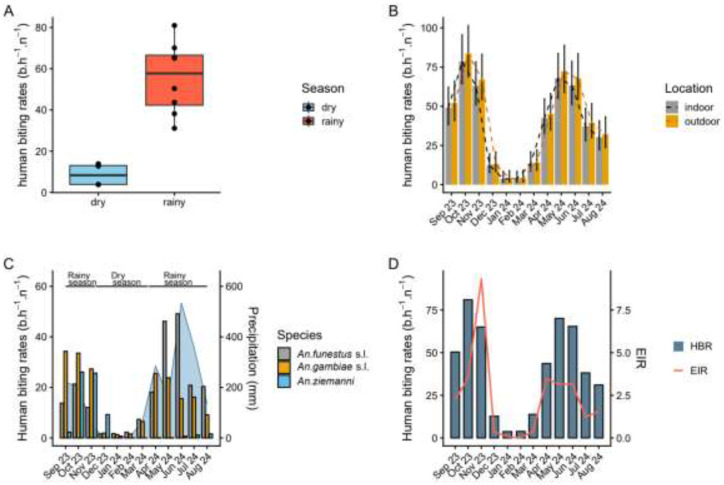
Seasonal variation of host-seeking behavior in collected *Anopheles* mosquitoes. **A:** Variation between seasons; **B**: Monthly variation between outdoor and indoor collection; **C**: Seasonal biting patterns of the collected *Anopheline* species and precipitation (blue shaded area); **D**: Monthly variation of the Entomological Inoculation Rate (EIR, dark orange line) in the mosquito population.

Out of 6,152 *Anopheles* collected, 51.7% (n=3,183, [95% CI: 50.0–53.5] were caught outdoors, and 48.3% indoors (n=2,969) [95% CI: 46.5–50.1; χ^2^=19.8, *p*<0.001]. This trend was consistent throughout the year (Fig. 3B). The higher proportion of outdoor bites was largely attributable to the exophilic behavior of *An. ziemanni* (59.8%, n=496/829).

The mean Human Biting Rate (HBR) was 39.9 bites/human/night (b/h/n), showing significant seasonal variation (Wilcoxon signed-rank test: Z= 5.63, *p=*0.004). The wet season HBR was 55.6 b/h/n [95% CI: 43.6–67.7], almost six times higher than the dry season 8.5 b/h/n [95% CI: 3.2–13.9] (Fig. 3A).

Indoor and outdoor HBRs were statistically similar (Wilcoxon signed-rank test: Z=3.45, *p=*0.712), with 41.2 b/h/n [95% CI: 24.8–57.4] outdoors and 38.7 b/h/n [95% CI: 23.8–53.6] indoors. Peak activity was observed in October, reaching 78.5 b/h/n indoors and 83.5 b/h/n outdoors (Fig. 3B).

Seasonal variation in biting rates differed among species. Human exposure to *An. funestus* s.l. bites were highest in May and June during the rainy season, peaking at 46.8 b/h/n in June. *An. gambiae* s.l. reached peak in September and October, averaging 34.3 b/h/n. *Anopheles ziemanni* populations exhibited a pronounced peak at the late rainy season, from October to December (26.1 b/h/n), with low biting rates observed during the dry season (Table 2, Fig. 3C).

The biting cycle indicated continuous *Anopheles* activity throughout the night, from 6 p.m. to 9 a.m. No significant differences were observed between indoor and outdoor bites for any species (*p*>0.9). However, peak biting times varied by species: *An. gambiae* s.l. exhibited peaks between 10 p.m. and 2 a.m. and *An. funestus* s.l. showed higher activity from 10 p.m. to 5 a.m. In contrast, *An. ziemanni* displayed a more uniform biting pattern throughout the night, with a plateau in activity from 10 p.m. to 6 a.m. (**Additional file**, **Fig. S2**).

### Parity rate and longevity

The overall parity rate for *Anopheles* mosquitoes was 76.6% (n=764/998; 95% CI: 73.9–79.2), indicating relatively high daily survival rates across the vector population. Although monthly rates varied, peaking at 100% during the mid-dry season (January) and reaching a low of 64.5% in the early rainy season (June), indicating a high proportion of older females from dry into rainy season, this variation was not statistically significant (ANOVA, F_1,11_ = 2.296, *p=*0.05). No significant differences in parity rates were observed among the three dominant species (*p=*0.53). (Table 2, **Additional file: Fig. S3**).

Using these parity rates, survival parameters were calculated. Both *An. funestus* s.l. and *An. gambiae* s.l. displayed comparable daily survival rates 0.92 ± 0.1 [95% CI: 0.86–0.97] and 0.91 ± 0.05 [95% CI: 0.88–0.94], respectively, resulting in long life expectancies of 12.0 and 10.8 days. Importantly, this longevity supports a substantial transmission potential, with an estimated 26% of *An. funestus* s.l. and 23% of *An. gambiae* s.l. surviving the minimum two weeks necessary for *Plasmodium* spp. infectious sporozoites to reside in the mosquito salivary glands. In contrast, *An. ziemanni* exhibited a similar daily survival rate of 0.93 ± 0.32 [95% CI: 0.64–1.03], but a slightly longer life expectancy of 13.6 days (X^2^ = 0.1, df = 2, *p=*0.95) (Table 2).

### *Plasmodium* Infection Rates and Seasonal Transmission Dynamics

Of the 2,291 *Anopheles* screened, 5.7% (n=131) were positive for *Plasmodium* spp. The infection rate (IR) was significantly higher in the rainy season (6.4%, n=120/1891) than in the dry season (2.5%, n=10/399) (χ^2^ = 8.4, df = 1, *p*<0.01).

*Anopheles funestus* s.l. displayed the highest IR at 7.8% (n=74/954, 95%CI: 1.7–13.9), followed by *An. gambiae* s.l. at 5.4% (n=50/937, 95%CI: −0.9–11.6), and *An. ziemanni* at 1.5% (n=6/399, 95%CI: −8.2–11.2). Seasonally, transmission was led by *An. funestus* s.l. in the rainy season with IR at 8.9% (n=71/800, 95% CI: 6.9–10.9), and *An. gambiae* s.l., in dry season with IR at 3.7% (n=5/136, 95% CI: 0.5–6.8). No significant variation in IRs was observed between indoor and outdoor settings (*p*>0.05) (**Additional file: Table S5**).

*Plasmodium falciparum* was the predominant parasite (70.2%, n=92/131), followed by *P. malariae* (27.5%, n=36/131) and *P. ovale* spp. (22.1%, n=29/131). While the parasite distribution among vectors was statistically similar (*p*>0.05), the temporal analysis revealed that while vector abundance (HBR) peaked in October (81 p/h/n), the infection rate reached its maximum in the last rainy season (November) at 14.3 % (n=43/300) (**Additional file: Fig S4**). The overall infectious burden was non-uniformly distributed (χ^2^ = 20.9, df = 2, *p*<0.0001). *An. funestus* s.l. harbored the majority of *P. falciparum* (60.3%, n=41/68) and *P. ovale* spp. (70%, n=14/20) mono-infections, while *An. gambiae* s.l. accounted for most *P. malariae* mono-infections (57.9%, n=11/19). *Anopheles ziemanni*, despite its lower abundance (13.5%), contributed to *P. falciparum* transmission but remained free of *P. ovale* spp. Mono-infections predominated, accounting for 81.7% (107/131), while *Pf/Pm* (63%, n=15/24) and *Pf/Po* (29%) were the most frequent co-infections (Table 3). No confirmed *P. vivax* infections were detected in any mosquito samples during the study period.

**Figure 4:**
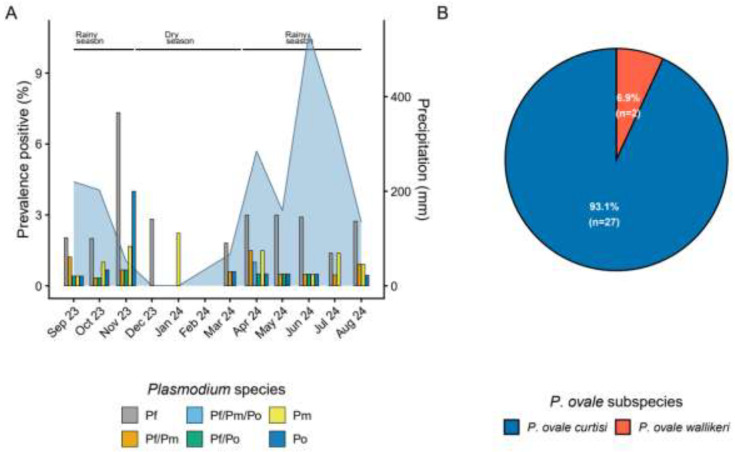
Prevalence of parasites in infected *Anopheles* mosquitoes collected. **A:** Monthly variation in parasite prevalence of infected *Anopheles* mosquitoes collected during this study period. The total monthly rainfall is denoted by the blue line (right Y-axis). **B**: prevalence of *Plasmodium ovale* subspecies in infected mosquitoes. *Pf* = *Plasmodium falciparum*, *Pm* = *Plasmodium malariae*, *Po* = Plasmodium ovale, *Pf/Pm* = *P. falciparum*/ *P. malariae*, *Pf/Po* = *P. falciparum/ P. ovale*, *Pf/Pm/Po* = *P. falciparum/ P. malariae/ P. ovale.*

Monthly parasite prevalence suggested temporally constrained transmission peaks (χ^2^ = 57.2, df = 11, *p*<0.0001). *P. falciparum* peaked at the end of the rainy season in November (13.5%), and peaked at 2.8% in the dry season (December). This occurred alongside a higher peak of *P. ovale* prevalence (4.0%), coinciding with maximum vector abundance (Fig. 4A). The circulation of *P. malariae* also showed significant peaks in the end of the rainy season in October (1.3%) and November (1.7%), followed by a second transmission peak during the dry season (January, 2.2%). Co-infections followed a distinct seasonal pattern linked to climatic transitions. Among the 29 *P. ovale* spp. infections (20 mono-infections and 9 co-infections), *P. ovale curtisi* predominated, accounting for 93.1% (27/29) of cases, while *P. ovale wallikeri* comprised 6.9% (2/29), and was detected only in *An. funestus* s.l. (Fig. 4B).

### Seasonal Entomological Inoculation Rates (EIR)

The overall entomological inoculation rate (EIR) was estimated at 2.3 infectious bites/human/night (ib/h/n), classifying this zone as a hyperendemic area (corresponding to an annual EIR of 839.5 ib/h/year). *P. falciparum* posed the highest risk infectious pressure (1.6 ib/h/n), followed by *P. malariae* (0.6 ib/h/n) and *P. ovale* sp. (0.5 ib/h/n). Transmission was primarily driven by *Anopheles funestus* s.l. (EIR of 0.9 ib/h/n), and *An. gambiae* s.l. (EIR of 0.6 ib/h/n), while *An. ziemanni* contributed minimally (0.05 ib/h/n).

The EIR displayed marked seasonality, peaking sharply during the rainy season in October (35.1 ib/h/n) and November (93.3 ib/h/n), led by *An. funestus* s.l., which exhibited a daily EIR of 1.4 ib/h/n. Conversely, the dry season showed minimal transmission, primarily led by *An. gambiae* s.l. (0.06 ib/h/n) (Fig. 3D). Comparison of biting locations revealed that outdoor EIRs were higher than indoors for both primary vectors (*p*<0.05). *Anopheles funestus* s.l. recorded 1.5 ib/h/n outdoors versus 1.3 ib/h/n indoors, while *An. gambiae* s.l. showed 1.1 ib/h/n outdoors compared to 0.7 ib/h/n indoors (**Additional file: Table S5**), suggesting that outdoor exposure represents the predominant inoculation risk.

### Frequency of insecticide resistance mutations

#### *Anopheles gambiae* s.l.

A total of 937 *An. gambiae* s.l. were screened for four key resistance mutations. The population exhibited high and complex patterns of resistance, often deviating from Hardy-Weinberg Equilibrium (HWE), indicative of strong selective pressure (Table 5, **Additional file: Fig. S5**).

The L1014F-*kdr* (West African *kdr*) mutation was highly prevalent with an allelic frequency of 89.8%. Genotypically, 81.4% (763/937) were homozygous resistant (RR), 16.8% (157/937) heterozygous (RS), and 1.8% (n=17/937) homozygous sensitive (SS), showing a significant deviation from HWE (χ^2^ = 6.7, *p=*0.03). Frequencies were stable throughout the year, with the highest rate during the dry season (98–100%) (Z= 1.9, df = 1, *p=*0.05).

The N1575Y mutation, which enhances the effects of *kdr*, was detected at a low allelic frequency of 6.1%. The population was predominantly sensitive with 88.1% (825/937) SS, 11.6% (109/937) RS, and only 0.3% (3/937) RR (χ^2^ = 0.09, *p=*0.96). Although its frequency appeared higher during the rainy season (6.8%) than in the dry season (2.9%), this difference was not statistically significant (χ^2^ = 2.6, df = 1, *p=*0.12).

The *E2025D-CYP6P3* mutation, associated with pyrethroid metabolic resistance, was observed at a high allelic frequency of 79.9%. The genotypic distribution was strongly skewed towards the resistant genotype: 65.9% (618/937) RR, 27.9% (261/937) RS, and 6.2% (58/937) SS. A significant deviation from HWE was also observed (χ2=16.7, *p=*0.002). Although the frequency was slightly higher during the rainy season (65%) compared to the dry season (59.2%), the difference was not statistically significant (χ^2^ = 2.1, df = 1, *p=*0.15).

The *G119S-Ace1* mutation associated with carbamate and organophosphate resistance was detected at a moderate allelic frequency of 49%. No homozygous resistant (RR) individuals were found, suggesting a potential fitness cost associated with the double- resistance genotype. The population consisted of 48.9% (459/937) RS and 51.0% (478/937) homozygous SS. This mutation exhibited a highly significant deviation from HWE (χ^2^ = 98.6, *p*<2.2e-16) and was the only tested mutation, showing significant seasonal variation (χ^2^ = 5.7, df = 1, *p=*0.02), rising sharply during the rainy season at 27.1% compared to the dry season (9.5%). Monthly variation was also significant, with frequencies peaking at 38% in July-August (*p*<0.001).

#### *Anopheles funestus* s.l.

Screening of 954 *An. funestus* s.l. specimens for three key resistance mutations (Table 4, **Additional file: S5**) revealed a marker of high metabolic resistance, with two of the screened mutations completely absent. The *L119F-GSTe2* mutation was detected at an allelic frequency of 55.7%. The population exhibited a genotypic distribution of 42.9% homozygous resistant (RR), 25.6% heterozygous (RS), and 31.6 % homozygous susceptible (SS). A highly significant deviation from HWE was observed (χ^2^= 221.5, *p*<2.2e-16), indicating a strong selective pressure on this locus. The mutation frequency was significantly higher during the rainy season (59.9%) compared with the dry season (33.8%) (χ^2^ = 5.6, df = 1, *p=*0.027). Temporally, the frequency peaked sharply in the early rainy season (April) at 73.5%, followed by a gradual decline toward the dry season, with a secondary peak in January (45.2%). However, monthly variation was not statistically significant (Z= 6.1, *p=*0.271). *CYP6P9a* and *CYP6P9b* mutations were not detected throughout the study period, with 100% of individuals identified as homozygous sensitive (SS) at both loci (Table 4).

#### Anopheles ziemanni

The secondary vector, *An. ziemanni* (n=399) was specifically screened for the L1014F-*kdr* mutation. The mutation was detected at a low allelic frequency (11.8%), and the population was predominantly sensitive, with 89.0% (355/399) homozygous SS, 8.5% (34/399) heterozygous (RS), and 7.5% (30/399) homozygous resistant (RR). A significant deviation from HWE was observed (χ^2^ = 138.9, *p*<2.2e-16) (Table 4).

No significant difference in mutation frequency was observed between the rainy and dry seasons (*p=*0.07). However, monthly variation was significant (Z= 4.3, *p*<0.001), with a pronounced allelic frequency peak in July (75%) (**Additional file: Fig. S6**).

### Influence of resistance markers on entomological parameters

**Figure 5:**
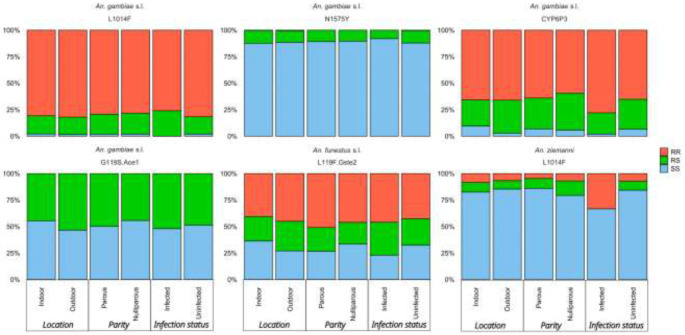
Influence of resistance markers on entomological parameters.

Analysis of the relationship between insecticide resistance genotypes and key entomological parameters revealed that while most markers were uniformly distributed, species- and locus-specific associations emerged across the *Anopheles* population studied (Fig. 5).

Regarding parity status, a significant association was exclusively observed in *An. funestus* (77.8%, n=347/446), where the *L119F-GSTe2* homozygous resistant (RR) genotype was dominant among parous females (50.1%, n=174/347; χ^2^ = 17.92, df = 4, *p=*0.001). In contrast, no significant link between age and resistance genotypes (CYP6P3, *L1014F-kdr, N1575Y*, or *G119S-Ace1*) was found for parous *An. gambiae* (75.8%; 332/438) or *An. ziemanni* (74.6%; n=85/114) (*p*>0.05), with mutations appearing equally distributed across age cohorts.

Biting behavior showed no significant stratification by resistance status. In all three species, genotype frequencies for both knockdown (*kdr*) and metabolic (*CYP6P3, GSTe2*) markers were nearly identical between indoor and outdoor collections (p>0.05). even in *An. funestus*, where *L119F-GSTe2* RR individuals showed a slight trend toward exophagy (23.1% vs 19.8%), this did not reach statistical significance (*p=*0.07).

**Figure 6:**
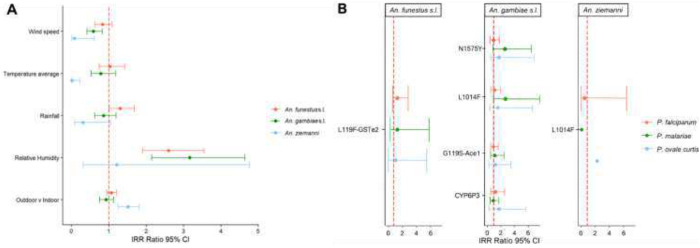
Generalized linear models (GLMs) exploring the association of **A**: climatic factor with species abundance; **B**: resistance mutations with *Plasmodium* species prevalence.

Finally, while resistance status did not significantly alter the overall probability of being infected (Fig. 6B), specific associations with *Plasmodium* species were identified through GLM analysis. Most notably, the presence of the *L119F-GSTe2* mutation in *An. funestus* accounted for the highest proportion of infections (45.9%, 34/74), and was associated with an increased likelihood of *P. ovale curtisi* infection (IRR = 1,15, *p=*0.03), suggesting a complex interaction between metabolic resistance and transmission dynamics (Fig. 6B; **Additional file: Table S8**). In *An. gambiae* s.l. mosquitoes (5.4%, *n=*50/937), homozygous resistant (RR) individuals for the major resistance markers (L1014F-*kdr* and *CYP6P3*) accounted for approximately 70% (39/50) of infections, with no significant differences were observed in infection distribution across genotypes for any individual mutation, indicating that although infections were concentrated among RR mosquitoes, resistance status itself did not significantly alter infection probability. A correlation was also noted between the L1014F-*kdr* mutation and a higher likelihood of *P. malariae* infection (IRR = 2,8, *p=*0,04). For *An. ziemanni*, 33% (2/6), no significant associations between *kdr* genotypes and *P. falciparum* prevalence were detected (Fig. 6B; **Additional file: Table S8**).

### Interplay between climatic variables, entomological parameters, and resistance mutations.

We observed a potential association of climate with mosquito population dynamics. Spearman correlation and GLMs consistently identified rainfall and relative humidity as the primary climatic drivers of mosquito abundance and transmission metrics. For example, *An. funestus* (r = 0.83, *p*<0.0001) and *An. gambiae* (r = 0.61, *p=*0.04) abundance showed a strong positive correlation with rainfall. Similarly, relative humidity was positively correlated with abundance for both *An. funestus* s.l. (r = 0.79, *p*<0.0001) and *An. gambiae* s.l. (r = 0.57, *p=*0.05). In contrast, *An. ziemanni* abundance exhibited a non-significant negative correlation with mean temperature (r = 0.54, *p=*0.07) (Table 5; **Additional file S6**).

Higher mosquito abundance was also significantly associated with increased malaria transmission potential, as reflected by positive correlations with the Human Biting Rate (HBR) (r = 0.68, *p=*0.02), Infection Rate (IR) (r=0.75, *p*<0.01), and Entomological Inoculation Rate (r = 0.7, *p*<0.0001). Poisson GLM analysis confirmed that relative humidity was a significant positive predictor of abundance for both species (*An. funestus*, IRR = 2.6, *p*<0.001; *An. gambiae*, IRR = 3.2, *p*<0.001). However, the influence of precipitation differed between species: monthly rainfall was significantly positive only for *An. funestus* -IRR = 1.3, *p=*0.05, whereas for *An. gambiae*, it showed a non-significant negative association (IRR =0 .85, *p=*0.36) abundance (Fig. 6A; **Additional file: Table S7**).

These data support an association of climatic factors and key insecticide resistance alleles. For *An. ziemanni*, higher temperatures and wind speeds were strongly associated with increased frequencies of the L1014F *kdr* allele (r = −0.9 and r = 0.8, respectively, *p*<0.001). In *An. gambiae* s.l., rainfall and relative humidity were significantly correlated with higher L1014F-*kdr* frequencies (r = −0.7, *p*<0.01 and r = −0.8, *p*<0.001, respectively). Conversely, rainfall showed a positive association with the L119F-GSTe2 metabolic resistance mutation in *An. funestus* (r = 0.7, *p*<0.01), while temperature and relative humidity were significantly correlated with the frequency of the G119S-*Ace1* mutation in *An. gambiae* (r = −0.9 and r = 0.8, respectively, *p*<0.0001) (Table 6).

## Discussion

The current study provides an integrated analysis of malaria transmission dynamics and insecticide resistance status of malaria vectors in a high-altitude setting in Western Cameroon (Penka-Michel).

The study revealed a complex, multi-species transmission system dominated by *An. funestus* s.l., followed by *An. gambiae* s.l. and *An. ziemanni*. Previous cross-sectional mapping at this site reported *An. gambiae* s.l. as the sole species in larval surveys, with adults collected in proximity to these temporary rain-fed pools^6,7^, and suggests a contemporary increase in the relative proportion of *An. funestus* s.l. during the collection period. Importantly. Vector abundance followed a clear seasonal pattern driven by rainfall and relative humidity, while *An. funestus* s.l. established early in the rainy season and persisted through the dry season, *An. gambiae* s.l. peaked later, coinciding with maximal availability of temporary breeding sites, and maintained its highest individual infection rate during the dry season, suggesting its important role in sustaining the parasite reservoir during low mosquito density periods. In contrast, *An. ziemanni* played a secondary but epidemiologically relevant role; its abundance was highest during the mid-to-late rainy season (October-November) and sustained into the transition period. Although less abundant, its continuous presence and its sustained parity are likely to contribute to low transmission continuity during this period, bridging seasonal gaps when primary vector populations fluctuate. This observation was streamlined by other highland studies, which reported *An. ziemanni* as a locally important vector in the highland areas of Cameroon^31^.

The entomological indices recorded indicate hyper-endemic transmission^32^, with a mean human biting rate (HBR) of 39.9 bites/human/night. Exposure concentrates during the rainy season, where biting rates increase six-fold. This intense exposure was largely due to vector behavioral plasticity^33,34^ of all three species, which exhibited mixed endophagic and exophagic biting behavior, with nearly equal proportions of indoor and outdoor biting. Peak biting activity occurred during late night and early morning hours, extending beyond periods when individuals are fully protected by insecticide-treated nets (ITNs)^35^. This behavioral flexibility critically undermines indoor-focused interventions and may partly explain residual transmission. Furthermore, high mean parity rates (>76%) observed reflect prolonged adult survival and favorable ecological conditions, allowing a substantial fraction of the vector population to exceed the *Plasmodium* extrinsic incubation period and sustain transmission into transitional seasons. The overall infection rate (5.7%) and daily EIR of 2.3 infected bites/human/year (corresponding to an annual EIR of 839.5 ib/h/yr) confirm Penka-Michel as a hyperendemic focus^32,36^, with strongly seasonal, rainfall-dependent transmission^37,38^. During the sampling time frame, *An. funestus* s.l. (IR: 7.8%) emerged as the principal vector for both *P. falciparum* and non-*falciparum* species. This contradicts earlier cross-sectional findings^6^, which described *An. gambiae* as the main, most abundant, and primarily infected vector; however, the discrepancy likely stems from sampling methodology. This longitudinal analysis captures the full annual cycle, revealing critical temporal variations in species composition and abundance that cross-sectional mapping often misses^39^.

The study confirms active transmission of multiple *Plasmodium* species. While *P. falciparum* remains dominant, consistent with the epidemiological profile of Cameroon and Sub-Saharan Africa^8,38^, *P. ovale curtisi* and *P. malariae* also significantly contribute to the parasite reservoir. A particularly notable finding is the observed peak in *P. ovale curtisi* (*Poc*) and *P. malariae* transmission occurring alongside *P. falciparum* at the end of the rainy season (November), a period that coincides with a climatic transition phase in the area, and the *P. malariae* transmission during the dry season (January). Moreover, when accounting for both mono and mixed infections, a secondary peak of *P. ovale* transmission was also observed in April, coinciding with the onset of the early rains. While this synchronized pattern in mosquitoes suggests that local ecological factors in the Cameroon highlands support the simultaneous transmission of both parasites, it contrasts with human prevalence data from Bagamoyo, Tanzania, where *P. ovale curtisi* and *P. malariae* peaks occur at times that differ from *P. falciparum* peaks^40^. Additionally, previous studies across various epidemiological settings within the country have reported significant transmission of *P. ovale curtisi* in both clinical and asymptomatic cases, indicating this species’ capacity to adapt to different zones^7,41,42^, and highlighting an overlooked, re-emerging threat in this malaria focus, possibly missed by current diagnostic tools. Considering its propensity to relapse, *P. ovale curtisi* can persist in human populations even when vector density is low, making elimination efforts more difficult.

Furthermore, the absence of confirmed *P. vivax*-infected vectors in the study site, despite systematic molecular screening, highlights a geographical variation in parasite distribution within the Western highlands. While clinical reports have identified *P. vivax* in nearby areas such as Dschang (0.1%, 2 cases on 3661 participants), the study findings revealed that Penka-Michel may function instead as a hotspot primarily for *P. ovale curtisi*^7^. This gap between regional clinical reports and our site-specific entomological data may be due to infection levels remaining below the threshold of molecular detection in the vector, or the specific ecological requirements for *P. vivax* transmission not being fully met at this study site.

Molecular analyses revealed multiple mechanisms of insecticide resistance across all vector species. In *An. funestus* s.l., resistance is characterized by the metabolic *L119F-GSTe2* mutation, which peaks during the early rainy season (April) under strong positive selection, establishing itself as the dominant local resistance mechanism^11,12^. The absence of *CYP6P9a/b* mutations suggests a locally specific resistance profile shaped by Penka-Michel’s agricultural highland landscape^43^. Intensive vegetable cultivation and the application of pesticides (such as the avermectin-class neurotoxic acaricide, Abalone^®^ 18 EC found in application in the field in this site) likely impose a strong cross-selection pressure, enhancing metabolic pyrethroid resistance by interaction with mosquito detoxification mechanisms^44^.

In *An. gambiae* s.l., resistance is high and multi-class. It’s driven by near-fixation of L1014F-*kdr* in the voltage-gated sodium channel (VGSC), a widespread genetic sweep primarily caused by the massive deployment of long-lasting insecticidal nets (LLINs) over the past few decades^12,45^, confirmed by a significantly high deviation from HWE observed in this study. This initial mutation is further amplified by the sustained presence of the synergistic N1575Y-*kdr* mutation (a second change in the VGSC located downstream of L1014F), which Jones et *al*.^26^ identified as a key enhancer that significantly boosts resistance to both pyrethroids and DDT. Compounding this target-site resistance is high metabolic resistance driven by the P450 enzyme CYP6P3; Kengne-Ouafo et *al*.^28^ specifically demonstrated that the single E205D allele of CYP6P3 is the primary driver of this metabolic defense, acting by enhancing insecticide breakdown. The combination of E205D-CYP6P3 and *kdr*, which leads to increased pyrethroid resistance, necessitates abandoning standard pyrethroid-based interventions in favor of next-generation tools, such as dual-active-ingredient LLINs, that can bypass these combined defenses. Furthermore, the presence of carbamate resistance (G119S-*Ace1*) in mosquitoes from this site poses an imminent threat, while currently limited by a probable fitness cost, its significant seasonal spike during the rainy season suggests concentrated selection pressure.

Carbamate insecticides must therefore be excluded as a replacement for pyrethroids in IRS to avoid accelerating their fixation in this locality^13,46^, and limit alternative insecticide options and argue against the use of carbamates in IRS programs^13^.

Interestingly, the secondary vector *An. ziemanni* also developed resistance to insecticides in Penka-Michel. While the main mosquito populations in Cameroon are largely sensitive, a sustained resistant sub-population carries the L1014F mutation of the VGSC gene, which, as reported by Mayi et *al*.^47^, suggests high selective pressure from agricultural pesticides.

Importantly, resistance does not merely prevent mosquito mortality; it may actively enhance transmission by promoting the survival of older, more infectious vectors, thereby profoundly undermining control efforts^11,14^. The study findings suggest a critical ecological trade-off. Specifically, the L119F-*GSTe2* mutation in *An. funestus* s.l. and L1014F in *An. gambiae* s.l. were slightly correlated with increased *P. ovale* and *P. malariae* infection, respectively. This may be explained by the oxidative stress mitigation hypothesis, where resistance mutations help mitigate the mosquito’s physiological stress, which is harmful to parasite development^11,14^. While these preliminary findings support the hypothesis of a metabolic trade-off enhancing vector competence, the statistical uncertainty observed (wide confidence intervals) calls for more extensive studies to confirm if resistance mechanisms are indeed reshaping the transmission landscape of *P. malariae* and *P. ovale*, creating a scenario in which insecticide pressure selects for more efficient vectors of relapsing malaria. Since resistance mutations showed no association with biting location, these vectors thrive both indoors and outdoors, undermining the effectiveness of indoor-only interventions. Furthermore, strong associations between climatic factors and resistance allele frequencies highlight the role of environmental selection in shaping resistance evolution.

## Conclusion

This study provides evidence that malaria transmission in Penka-Michel is perennial, hyper-endemic, and ecologically complex, driven by seasonally structured vector dynamics and exacerbated by widespread insecticide resistance. *Anopheles funestus* s.l. is likely the primary transmission vector during the study period, sustaining both *P. falciparum* and *P. ovale curtisi*, while *An. gambiae* s.l. and *An. ziemanni* both act as important complementary vectors. Beyond climatic drivers like rainfall and humidity, this study reveals a concerning landscape of diverse resistance mutations, notably *L119F-GSTe2* in *An. funestus* s.l. and *L1014F*-*kdr*, *N1575Y*-*kdr*, *CYP6P3*, and *G119S-Ace1* in *An. gambiae* s.l. poses a major threat to current vector control tools. Currently, a possible association between specific resistance markers and increased carriage of non-*falciparum* parasites has not been fully explored. However, our findings should be interpreted with caution.

These findings highlight the urgent need for integrated, climate-informed, and locally adapted vector control strategies, including insecticide rotation, next-generation LLINs, outdoor interventions, and continuous molecular surveillance. Without such adaptive approaches, insecticide resistance and climatic variability will continue to undermine malaria control efforts in the western highlands of Cameroon.

## Supplementary Material

**Supplemental Information:** the online version contains supplementary material available at (html link)

Supplementary Files

This is a list of supplementary files associated with this preprint. Click to download.

- SupplementaryFileComplexnonfalciparumtransmissionandwidespreadinsecticideresistanceinPenkaMichelahyperendemicmalariafocusoftheCameroonhi

## Figures and Tables

**Table 1:** Insecticide resistance markers genotyped in collected *Anopheles* mosquitoes.

Mosquito species	Resistance marker	Associated Insecticide	Genotyping method	Reference
*An. gambiae* s.l.	L1014F-*kdr*	Pyrethroids, DDT	PCR assay / TaqMan assay	^25^
	N1575Y-*kdr*	Pyrethroids, DDT		^26^
	G119S-Ace1	Carbamates, Organophosphates	TaqMan assay	^27^
	E205D-CYP6P3	Metabolic resistance (pyrethroids)	PCR assay	^28^
*An. funestus* s.l.	L119F-GSTe2	Metabolic resistance (DDT, pyrethroids)	TaqMan assay / PCR-RFLP	^29,30^
	CYP6P9a	Metabolic resistance (Pyrethroids)	TaqMan assay / PCR-RFLP	
	CYP6P9b	Metabolic resistance (Pyrethroids)	TaqMan assay / PCR-RFLP	
*An. ziemanni*	L1014F-*kdr*	Pyrethroids	PCR assay	^25^

**Table 2:** Parity rate and longevity parameters of anopheline species in the study site.

	*An. funestus* s.l.	*An. gambiae* s.l.	*An. ziemanni*
Human biting rate (ma)	11.2	10.9	3.5
Parity rate (%)	77.8	75.8	74.6
Daily survival-p^1^	0.92	0.91	0.93
p^16^ (%)	26	23	31
Life expectancy^2^	11.95	10.83	13.62

1 Daily survival (p) was taken as ^3^√*P* (*P*=percentage parous)^48^

2 Life expectancy = 1/−log_e_ *p* (days)^3^

p^16^ Percentage of population expected to live long enough to become infective with an extrinsic cycle of 16 days based on *Plasmodium* spp. extrinsic incubation period calculated as EIP=111/Tmean-16

Tmean=22.84°C (mean of daily temperature from September 2023 to August 2024) https://power.larc.nasa.gov/data-access-viewer/

**Table 3:** *Plasmodium* infection by anopheline species.

Species		*An. funestus* s.l. (N=954)		*An. gambiae* s.l. (N=937)		*An. ziemanni* (N=399)		Total (N=2,290)		*p*-value^a^
		n	%	n	%	n	%	n	%	-
Uninfected		880	40.7	887	41.1	393	98.49	2,160	94.3	
Infected	*Pf*	41	60.3	23	33.8	4	5.9	68	51.9	0.02*
	*Pm*	7	36.8	11	57.9	1	5.3	19	14.5	ns
	*Po*	14	70	6	30	-	-	20	15.3	ns
	*Pf/Pm*	8	53.3	6	40	1	6.7	15	11.5	ns
	*Pf/Po*	3	42.9	4	57.1	-	-	7	5.3	ns
	*Pf/Pm/Po*	2	100	-	-	-	-	2	1.5	-
	Total	75	56.5	50	38.2	6	4.6	131	5.7	

a p-value adjusted for multiple comparisons using the Bonferroni method

ns= not significant; N= population size; n= sample size; %= percentage

**Table 4:** Allele frequency and distribution of insecticide resistance in the *Anopheles* vector collected.

Species	Resistance mutations	Mosquitoes tested	Homozygous resistant (RR)	Heterozygous (RS)	Homozygous sensitive (SS)	Allelic frequency (%)		HWE x2 test	*p*-value
						R	S		
*An. gambiae* s.l.	L1014F	937	763	157	17	89.8	10.2	6.7	0.03
	N1575Y	937	3	109	825	6.1	93.9	0.09	0.9
	G119S-Ace1	937	0	459	478	49.0	51.0	98.6	<2.2e-16
	CYP6P3	937	618	261	58	79.9	20.1	16.7	<0.001
*An. funestus* s.l.	L119F-GSTe2	954	409	244	301	55.7	44.3	221.5	<2.2e-16
	CYP6P9a	954	-	-	954	-	100	-	-
	CYP6P9b	954	-	-	954	-	100	-	-
*An. ziemanni*	L1014F	399	30	34	335	11.8	88.2	138.9	<2.2e-16

HWE = Hardy-Weinberg Equilibrium

**Table 5:** Spearman’s correlation (r^2^) of species *Anopheles* with climatic variables and their significant *p*-value.

Species	Rainfall		Temperature		Relative humidity		Wind speed	
	r^2^	*p*-value	r^2^	*p*-value	r^2^	*p*-value	r^2^	*p*-value
*An. funestus* s.l.	0.8	<0.0001*	−0.4	0.22	0.8	<0.0001*	−0.1	0.83
*An. gambiae* s.l.	0.6	0.04*	−0.2	0.51	0.6	0.05*	−0.2	0.5
*An. ziemanni*	0.1	0.97	−0.5	0.07	0.3	0.41	0.3	0.43

**Table 6:** Influence of climatic variables on insecticide resistance in *Anopheles* collected.

	Resistance mutations	Rainfall		Temperature		Relative humidity		Wind speed	
Species		r^2^	*p*-value	r^2^	*p*-value	r^2^	*p*-value	r^2^	*p*-value
*An. funestus* s.l.	L119F-GSTe2	0.7	0.01*	−0.2	0.63	0.5	0.11	−0.4	0.2
*An. gambiae* s.l.	L1014F	−0.7	0.01*	0.3	0.33	−0.7	<0.001*	−0.2	0.68
	N1575Y	0.5	0.1	−0.1	0.72	0.4	0.19	0.3	0.31
	G119S-Ace1	0.5	0.1	−0.9	<0.0001*	0.8	<0.0001*	0.4	0.15
	CYP6P3	−0.1	0.66	0.2	0.62	0.1	0.94	0.1	0.72
*An. ziemanni*	L1014F	0.3	0.35	−0.9	<0.001*	0.6	0.07	0.8	<0.001*

## Data Availability

Meteorological datasets used in this study were obtained from the NASA Langley Research Center POWER Project via the NASA POWER portal (https://power.larc.nasa.gov/data-access-viewer/). Specific parameters include (cumulative daily precipitation, daily average relative humidity, daily temperature (average, maximum, and minimum), and average wind speed). The other datasets generated or analyzed during this study are included in this published article and its supplemental files.
